# Assessing the gain of biological data integration in gene networks inference

**DOI:** 10.1186/1471-2164-13-S6-S7

**Published:** 2012-10-26

**Authors:** Fábio FR Vicente, Fabrício M Lopes, Ronaldo F Hashimoto, Roberto M Cesar

**Affiliations:** 1 Federal University of Technology - Paraná, Brazil; 2 Institute of Mathematics and Statistics, University of São Paulo, Brazil; 3 Brazilian Bioethanol Science and Technology Laboratory (CTBE), Brazil

## Abstract

**Background:**

A current challenge in gene annotation is to define the gene function in the context of the network of relationships instead of using single genes. The inference of gene networks (GNs) has emerged as an approach to better understand the biology of the system and to study how several components of this network interact with each other and keep their functions stable. However, in general there is no sufficient data to accurately recover the GNs from their expression levels leading to the curse of dimensionality, in which the number of variables is higher than samples. One way to mitigate this problem is to integrate biological data instead of using only the expression profiles in the inference process. Nowadays, the use of several biological information in inference methods had a significant increase in order to better recover the connections between genes and reduce the false positives. What makes this strategy so interesting is the possibility of confirming the known connections through the included biological data, and the possibility of discovering new relationships between genes when observed the expression data. Although several works in data integration have increased the performance of the network inference methods, the real contribution of adding each type of biological information in the obtained improvement is not clear.

**Methods:**

We propose a methodology to include biological information into an inference algorithm in order to assess its prediction gain by using biological information and expression profile together. We also evaluated and compared the gain of adding four types of biological information: (a) protein-protein interaction, (b) Rosetta stone fusion proteins, (c) KEGG and (d) KEGG+GO.

**Results and conclusions:**

This work presents a first comparison of the gain in the use of prior biological information in the inference of GNs by considering the eukaryote (*P. falciparum*) organism. Our results indicates that information based on direct interaction can produce a higher improvement in the gain than data about a less specific relationship as GO or KEGG. Also, as expected, the results show that the use of biological information is a very important approach for the improvement of the inference. We also compared the gain in the inference of the global network and only the hubs. The results indicates that the use of biological information can improve the identification of the most connected proteins.

## Background

The regulation of a diversity of biological process is only possible because of the interaction between distinct components [1], maintaining the homeostasis of the system. A major challenge in biological sciences is to understand how several components interact with each other in order to perform their functions. By considering that proteins play their action not alone but into the context of a network of interactions [2], the mapping of the interrelationships among proteins is an important step to understand their functions and the global cell behavior [3]. In recent years, new high-throughput technologies allowed the measuring of expression profiles of thousands genes simultaneously. Because of the large amount of transcriptome data available, the inference of gene networks (GNs) from expression data has emerged as an approach to the study of the systems biology [4]. The assumption is that if there is an interaction between two elements (e.g. protein-protein, Transcription Factor-DNA, etc.) their expression profiles should also be related. However, two genes may have similar expression profiles just by coincidence. Thus, the challenge is to recover GNs reducing the number of false positives. The expression data can be sampled as time points (time-series / time-course data) or under different biological conditions (steady state data). Also, the data can be produced by distinct technologies as microarrays [5], SAGE [6] and RNA-Seq [7].

The so-called "curse of dimensionality" [8] is a phenomenon in which the number of training samples required for a satisfactory classification is given by an exponential function of the size of the feature space. In many applications, and especially in systems biology, the size of the training samples is generally much lower than the dimension of feature space. Thus, despite high-throughput data available, there is still a limitation in the inference of GNs: the number of genes (features) is much larger than the number of time-points (samples). As an example the expression dataset of *P. falciparum *has 7,745 oligos and only 48 time points.

Facing this problem, other biological information than expression data has been included in order to reduce the estimation error [9,10]. Several types of new biological data have been recently produced: (a) Interaction Data: protein-protein interaction [11-13] and protein-DNA [14], (b) Function and Ontology: KEGG [15] and Gene Ontology [16], (c) Other like phylogenetic profile [17] and Rosetta stone fusion proteins [18,19]. In a recent work [20], pairwise relationships obtained from Gene Expression, Phenotypical Profile, KEGG Pathway, Transitive Homology of protein sequences and protein-protein interaction were used to increase the positive predictive value (PPV) [21] and to predict the gene function. Each dataset comprises pairwise relationships between genes and each pair has an associated similarity measure (except in protein-protein dataset). A PPV is calculated for each dataset at each similarity value using Yeast Gene Ontology annotation as Gold Standard. After, the PPVs were combined into an equation (Biological Score) and weights were associated to each PPV. Gene pairs were grouped according to the Biological Score using the KNN cluster algorithm [22]. Gene function was associated to genes according to the group.

Although several data sources are integrated and increase PPV, the result is only related to Gene Ontology and the gain of adding each information remain unclear. The work [20] is based on the assumption that if two genes are related in the information dataset they should share a common GO term. In other words, the weights show how much each information contributes to recover Gene Ontology relationships but they do not make clear how each information contributes to recover the same type of information itself. Other study [3] assessed the limit of data integration to predict protein-protein relationships. Using a Bayesian classifier, the relationship between the number of features (prior biological information) and the improvement in the predictive power were evaluated. An improvement in accuracy and coverage was achieved by integrating data of four strongest features: a) functional similarity based on GO, b) functional similarity based on MIPS Functional Catalog database, c) coessentiality and d) correlation between expression data. The MIPS Functional Catalog is a database of protein function provided by the Munich Information Center for Protein Sequences (MIPS) [23]. The MIPS terms are arranged hierarchically according to classes (e.g. 01:Metabolism, 01:04 phosphate metabolism, 01:04:04 regulation of phosphate metabolism, 02.Energy, etc.). Data from GO and MIPS are important because proteins that belong to same biological process are more likely to interact [3].

Also, statistical dependence between features (types of biological information) was analyzed. The absence of statistical dependence between the features available was another important discovery.

Although increasing performance in prediction of protein-protein interactions is very significant, some other important aspects must be highlighted: (a) The prediction was not done in the context of the inference of GNs from expression data, (b) the gain of each information is unclear, (c) the protein-protein itself as prior information was not evaluated.

Another important aspect is that several approaches of data integration are based on the correlation measure between expression profiles of gene pairs in biological data (like protein-protein networks) as in [20]. However, it has been shown that opposite to prokaryotes organisms, in eukaryotes the correlation of related pairs is similar to those of random networks [24]. Also, [25] showed that transient protein complexes have a weak correlation to expression profiles. Other work [26] observed that although in *S. cerevisiae *and *bacteriophage T7 *co-expression and protein-protein interaction are related, self-interactions cannot be tested by correlation between expression profiles which notably represent a considerable proportion of the samples. In this work we address this problem by using an approach based on mean conditional entropy. Previous works [9,10,27] in data integration have contributed to decrease the estimation error. However, some questions are still unclear:

*What is the gain of adding different biological information in the GNs inference? *In a previous work [28] we describe the gain of adding protein-protein data to recover the GN of *Plasmodium falciparum*. Also, added data of distinct types are normally evaluated against a common gold standard related to a single feature (functional, physical contact, etc.). It is not clear what is the gain to infer the same type of data added. For example, *what is the gain of adding protein-protein information to recover a protein-protein network?*

An important aspect of adding biological information is the heterogeneity of data.

For example, protein-protein networks obtained from Yeast two hybrid experiment (Y2H) are *in vitro *verification of physical interaction. Rosetta stone fusion pairs are prediction of protein-protein interactions obtained indirectly through sequence comparison. KEGG data can inform if two components interacts in the same pathway. The Gene Ontology (GO) information is useful to obtain the physical cellular localization, biological process or molecular function. In general, it does not make clear if genes sharing the same GO-term interact directly with each other. Thus, we propose a classification into two types of biological information data: (a) direct physical interaction data (e.g. protein-protein, protein-DNA) and (b) feature data (e.g. biological process, cellular localization, signaling pathway in which participates, etc.).

Thus, another important question is: *what is the relative gain of distinct types of biological information? *It is not clear if they have the same behavior.

In this work we developed an algorithm to integrate biological information data for the inference of GNs, evaluated and compared the relative gain of four biological information dataset for the *P. falciparum *organism: (a) Protein-protein interaction, (b) Rosetta Stone fusion proteins, (c) KEGG, (d) Combined KEGG and GO dataset.

## Methodology

An important aspect regarding on introduction of a new algorithm for the inference of GNs is to evaluate whether or not the inferred relationships are reliable. In order to do such assessment it is necessary to evaluate its performance by using simulated or real biological data.

By considering biological data, the evaluation of predicted interactions can be carried out through a gold standard dataset. However, there is not a consensus about how a gold standard dataset should be constructed. Particularly, in the inference of GNs a gold standard can mean genes on the same metabolic or signaling pathway, genes sharing the same ontology terms, protein-DNA interaction, protein-protein interactions, etc.

Thus, a common approach to evaluate predicted links between genes is to questioning if each inferred relationship is supported by some annotation data. Such evidence can be obtained if the predicted gene pair: (a) participates in the same pathway, (b) shares the cellular localization or (c) has the same gene function. Such data is a type of qualitative information that does not make clear if the two genes (or their corresponding products) have a physical interaction. However, the annotation data allows counting predicted pairs that are biologically related.

Another way to evaluate the predicted network is by counting the number of predicted links corresponding to real physical contact like protein-protein interactions.

In this work we assembled one main Sample Expression Dataset and four Gold Standard datasets: (a) protein-protein, (b) Rosetta Stone fusion, (c) KEGG, (d) KEGG+GO. For each gold standard a sample expression dataset was assembled containing only the subset of genes in the gold standard set resulting in four sample expression datasets, one for each gold standard. Thus, each analysis was performed for a sample and the corresponding gold standard network.

### Biological dataset - expression data

The *Malaria expression dataset *used in this work was first reported by [29]. The dataset consist of 48 time samples of 7,745 genes of *P. falciparum *HB3 strain. We used preprocessed samples acquired from USP dataset in [30]. The dataset comprises only genes that were filtered through a quality control (leave out genes with less than 25 time points sampled) resulting in samples of 6,532 genes (Additional file 1: Table S1). Since the algorithm deals with discrete data, the original expression dataset (real numbers) of [29] was normalized and quantized into three levels (-1,0,+1): down, basal and up regulated in relation to the reference in order to fit the algorithm (Additional file 2: Table S2 and Additional file 3: Table S3).

As developed in [30], the expression time series of each gene *g*(*t*), *t *= 1, 2, .... *n*, are normalized by the *normal transformation*:

(1) $$\eta \left[g\left(t\right)\right]=\frac{g\left(t\right)-E\left[g\left(t\right)\right]}{\sigma \left[g\left(t\right)\right]}$$

where *σ*[*g*(*t*)] is the standard deviation of *g*(*t*) and *E*[*g*(*t*)] is the expectation of *g*(t).

After the *normal transformation*, the signal *α *of every gene *g *at a each instant *t *≥ 0 is quantized through a *threshold mapping*:

(2) $$g\left(t\right)=\left\{\begin{array}{cc} +1\; & \alpha \geq h\\ 0\; & l\leq \alpha \leq h\\ -1\; & \alpha \leq l \end{array}\right)$$

Also, in each subsequent analysis, only samples in both expression dataset and in the gold standard network were selected. Samples that were not present in the gold standard were excluded.

### Protein-protein network

We used *P. falciparum *protein-protein network identified by LaCount [13] through a high throughput version of yeast two-hybrid assay as gold standard. Briefly, that network was generated using RNAs isolated from mixed intraerythrocytic stage that were used to build the libraries of two-hybrid assay. The resulting data lack genes expressed exclusively in the liver, gametocyte and mosquito stage. In order to obtain a reliable network, several interactions obtained from 32,448 yeast two-hybrid screens were eliminated in a downstream analysis. Protein fragments with many partners were eliminated to avoid promiscuous interactions in the two-hybrid assay. The resulting data is a highly interconnected scale-free network comprising 1,267 proteins (vertex) and 2,823 interactions (edges).

We selected the subset of genes present both in LaCount protein-protein network and Expression Profiles. Our resulting gold standard comprises 1,958 interactions between 985 genes (Additional files 4 and 5: Tables S4 and S5 respectively).

### Rosetta Stone

Rosetta Stone protein data represents prediction of protein-protein interactions inferred from genome sequences. The information of this type of data is similar to that from Y2H experiments and are related to physical interactions.

The term "Rosetta Stone" was proposed by [18] and is based on the observation that some proteins that participates in a protein-protein interaction in an organism forms a single protein chain in another organism. In other words, sequences of two interacting proteins in one organism are homologous to a single protein in another organism. Thus, this association is a "Rosetta Stone" that "deciphers" the protein-protein interaction.

In this work we used the dataset of Rosetta stone fusion proteins developed by [31]. Briefly, it was used *Plasmodium falciparum *plus 164 other genomes and the presence of Rosetta stone fusion proteins was verified. It was excluded links for which fusion proteins were found only in *Plasmodium*. Thus, 993 proteins with 5,176 links compose the original dataset.

We selected proteins found both in the Rosetta stone fusion proteins network and in the Expression Dataset. The resulting network comprises 2,146 links between 702 genes (Additional files 6 and 7: Tables S6 and S7 respectively).

### KEGG pathway dataset

The Kyoto Encyclopedia of Genes and Genomes (KEGG) pathway [15] is a description of several molecular interactions and how they are organized in a biological pathway. KEGG data is a rich source of biological information and at least two types of information can be obtained: (a) if two gene products interact each other directly and (b) if two gene products interact each other indirectly into the same pathway.

The KEGG gold standard is a set of KEGG association obtained from the work of Bozdech [32]. The dataset comprises 11,046 links between 492 genes. After filtering genes found in both expression and KEGG datasets the resulting network comprises 393 genes with 8,750 links (Additional files 8 and 9: Tables S8 and S9 respectively).

### Combined KEGG and GO network dataset

The Gene Ontology (GO) (Gene Ontology Consortium 2004) [33] is a source of information about three point of views: cellular component (the physical localization in the cell or its extracellular environment), molecular function and biological process. Thus, once a gene pair is associated through some method it is possible to argue which the two elements share the same GO term. Also, it is important to know at what *level *(in GO structure) two genes shares the same annotation. For example, suppose that the gene pair is associated to the GO term *cellular metabolism*. Can we use this information to accept the relationship? There are several components involved on *cellular metabolism *and they are not all directly related to each other. Thus, depending on the type of study the data could provide a "poor" information. We could found another scenario in which the data could be "richer". For example, if the genes were both associated to *acetyl-CoA biosynthesis from acetate*. This is a more specific level of metabolism than *cellular metabolism*. Thus, this type of biological data does not make clear if the two components interact directly with each other. However, it can provide an idea of how much a predicted link is reliable. In this work we used information of a valuable dataset of KEGG pathways and biological process annotation (GO) data developed by [31]. The dataset consists on preprocessed and combined information of the KEGG pathways and GO of *P. falciparum*. In the preprocessed dataset a minimum GO term depth of 5 was maintained. The dataset contains 10,267 links between 412 proteins. We filtered genes that were not in our Expression Dataset resulting in a network containing 344 proteins and 7,204 links (Additional files 10 and 11: Tables S10 and S11 respectively).

### Overlap between gold standard datasets

The presented gold standard data differs in size, genes and edges. As shown in Table 1, the networks with smallest number of genes (KEGG and GO) have the largest number of edges (this points to high connected networks) while the gold standard Protein-Protein has the largest number of genes and the smallest number of links. This indicates that available dataset may present different topological features (due the sampling). Also, the datasets can have different genes and links.

**Table 1:** Summary of the 4 gold standard datasets.

Gold Standard	Genes	Links	Reference
Protein-Protein	985	1958	LaCount et al, 2005
Rosetta Stone	702	2146	Date et al, 2006
KEGG	393	8750	Bozdech et al, 2003
KEGG+GO	344	7204	Date et al, 2006

We analyzed the overlap between the four gold standards. A summary of the comparison is shown in Table 2. A low percentage of Protein-Protein genes is found in other networks: 240 of 985 (24%) in Rosetta Stone, 11% in KEGG and only 8.7% in KEGG+GO. The percentages of Rosetta Stone genes present in Protein-Protein, KEGG and KEGG+GO are respectively 34%, 15% and 14%. There is a very low overlap of edges, especially in Protein-Protein data.

**Table 2:** Overlap between the 4 gold standard datasets.

	Rosetta Stone		KEGG		KEGG+GO	
	**Genes**	**Links**	**Genes**	**Links**	**Genes**	**Links**

Protein-Protein	240	4	113	4	86	3
Rosetta Stone			107	45	105	28
KEGG					316	6,138

Genes and links was counted separately. In other words, a gene was counted if it was found in both dataset even without edges.

KEGG and KEGG+GO datasets present the higher overlap. In fact, there are 316 common genes of 393 and 6,138 edges of 8,750. This suggest a minor contribution of GO data on this available dataset although GO is a rich source of information.

### Intersection gold standard dataset

Due to the heterogeneous nature of different datasets, we explore the possibility of using the intersection of the datasets as a high quality gold standard. An "intersection dataset" is interesting because the gain (of each independent dataset) can be evaluated in respect to a common gold standard, and thus could help avoiding a possible bias. However, as shown in the previous section, it is not possible to combine all datasets because there is not a large overlap between their edges. This points to a practical limitation in the evaluation of improvement of adding data information: in general the available data come from different and heterogeneous sources.

Despite these limitations we propose here four Intersection Gold Standard datasets (IGS) as shown in Table 3. The Protein-Protein network was left out since it has not intersection (intersection of only four edges) with other networks. These assembled network are smaller than each original gold standard but it captures the relationships observed in all networks.

**Table 3:** Summary of 4 Intersection Datasets.

IGS	Intersection Gold Standard	Genes	Links
IGS1	Rosetta ∩ KEGG +GO ∩ KEGG	25	21
IGS2	Rosetta Stone ∩ KEGG	31	45
IGS3	Rosetta Stone ∩ KEGG +GO	36	28
IGS4	KEGG ∩ KEGG + GO	314	6,138

### Inference of gene networks

In order to recover the GNs from expression profiles, it was applied the approach described in [34], which is based on a feature selection algorithm. A feature selection method is commonly composed of two parts: a search algorithm and a criterion function that attributes a quality value to the feature subsets, in order to select a subset of features that makes a good representation, classification or prediction of states (or values) of the objects in study.

As explained before, in gene networks inference, the number of genes is generally much larger than the number of time-points. In this context, the investigation of effective ways of data integration in the inference methods represents an important challenge in systems biology research.

The data integration approach considered in this work is described in [20], which proposes a biological score (BS) as a weighted sum of available biological information. In this work, it is proposed a criterion function inspired on BS, which is defined as follows:

(3) $$BS_{Y,X}=E_{Y,X}\times w_{1}+P_{Y,X}\times w_{2},$$

in which given a fixed gene *Y *as target, the general idea of this model is to determine the subset of genes **X **that makes the best prediction of the *Y*, by taking into account *E *and *P*. *E *is the penalized mean conditional entropy score obtained from expression profiles [34] and *P *is the information about protein-protein interaction.

The mean conditional entropy is based on the distributions between classes of conditional probability of the target gene given the subset of predictors, i.e., the more concentrated is the conditioned distribution on the observation of the predictors, the lower is the corresponding entropy.

In this work, the coefficients *w*_1 _and *w*_2 _are positive real numbers such that $\sum_{w_{i}}=1$, from which one needs to find a value combination that maximizes the predictive power between a target gene and its predictors. In order to clarify how the GNs inference algorithm works, it is presented in Algorithm 1 its pseudo code.

Initially, the algorithm receives its parameters, in which *target *represents the target gene identification for the inference of its predictors and the *kmax *representing the maximum cardinality of the subset of predictors that will be searched. The *w*_1 _and *w*_2 _are weights for the information of the expression profiles (*Expressions*) and the biological interaction data like protein-protein interaction (*BiologicalInteraction*), respectively. After that, the SFFS Algorithm [35] is applied in order to discover the best *newsubset *of predictor genes for the target by considering the cardinality (*k*), in terms of the adopted criterion function, i.e., Eq. 3. The variable *newvalue *represents the criterion function value achieved by the *newsubset *of predictor genes. If the *newvalue *presents better results than *bestvalue*, then the *bestvalue *and *bestset *are updated by *newvalue *and *newsubset*, respectively. The *while *loop performs the SFFS algorithm for each cardinality *k *= 1, ..., *kmax *trying to find the best subset of predictors in each cardinality *k *keeping the best global solution in the variables *bestvalue *and *bestset*. When its cardinality achieves the limit, i.e., *kmax*, the inference algorithm is stopped and returns the best subset of predictors achieved for the target gene.

### Validation of the information gain

In order to quantify the similarity between the gold-standard and inferred networks, it was adopted the PPV (Positive Predictive Value, also known as accuracy or precision) and Sensitivity (or recall) measurements presented by [21], which are based on a confusion matrix [36] and widely used to compare the results of the gene inference methods.

As presented in [37], the confusion matrix measures could be interpreted as how much the GNs inference method gets "confused" in inferring the network edges. The measures considered in this work are based on topological features, which are described in the confusion matrix showed in Table 4. These measures contain information about the correct and incorrect inferred edges by considering a gold-standard network and an inferred network. The measures in this confusion matrix have the following meaning in the context of this work: TN is the number of non-identified edges that are absent in the gold-standard network, FP is the number of identified edges that are absent in the gold-standard network, FN is the number of non-identified edges that are present in the gold-standard network, and TP is the number of identified edges that are present in the gold-standard network.

**Table 4:** Confusion matrix.

Edge	Inferred	Not Inferred
Present	TP	FN
Absent	FP	TN

TP = true positive, FN = false negative, FP = false positive, TN = true negative.

**Algorithm 1 **GNsInference (*target*, *kmax*, *w*_1_, *w*_2_, *Expressions*, *BiologicalInteraction*)

1: **var ***list bestset, newsubset*

2: **var ***float bestvalue, newvalue*

**3: var ***integer k *← 1

4: **while ***k *≤ *kmax ***do**

5:      [*newsubset*, *newvalue*] ← SFFS (*target*, *w*_1_, *w*_2_, *Expressions*, *BiologicalInteraction*, *bestset*, *k*)

6:      **if ***newvalue < bestvalue ***then**

7:         *bestvalue ← newvalue*

8:         *bestset ← newsubset*

9:      **end if**

10:      *k *← *k *+ 1

11: **end while**

12: **return ***bestset*

In this approach, the networks are represented in terms of their respective adjacency matrices *M*, such that each edge from node *i *to node *j *implies *M*(*i*, *j*) = 1, with *M*(*i*, *j*) = 0 otherwise. The measures considered in this work are calculated as follows:

(4) $$\begin{array}{c} Similarity\left(A,B\right)=\sqrt{PPV\cdot Sensitivity,}\\ PPV=\frac{TP}{\left(TP+FP\right)},\;Sensitivity=\frac{TP}{\left(TP+FN\right)}. \end{array}$$

By observing the ground truth network *A *and the inferred network *B*, the measure Similarity(*A, B*) is the geometrical average between the ratios of correct and incorrect inferred edges, implying that the maximum similarity to be obtained for values near 1. In order to analyze the gain each measures was normalized into the interval (0, 1).

We performed four distinct evaluations of quantitative gain in respect to each gold standard dataset. Thus, in each evaluation, a gene network was inferred from the expression profiles of the corresponding genes in the selected ground truth. Instead of adding distinct biological information and comparing to a unique common gold standard we used the information added as the ground truth network. Thus, we verified the gain of each type of information in respect to the expression profile information. In other words, the variation of biological information weight can give an idea of the relationship between expression profiles and the biological data.

## Results

This section describes the experimental results obtained by considering distinct biological information as well as gold-standard networks and the inferred networks from two distinct situations: from temporal expression profiles and by biological score approach, which in this work was obtained by combining expression profiles and biological data.

The same method and parameters (default) were kept fixed during comparative analysis. It is important to notice that temporal data is considered for the inference method in a dynamical way, such that auto-relationships were not considered in the present work.

**Figure 1:**
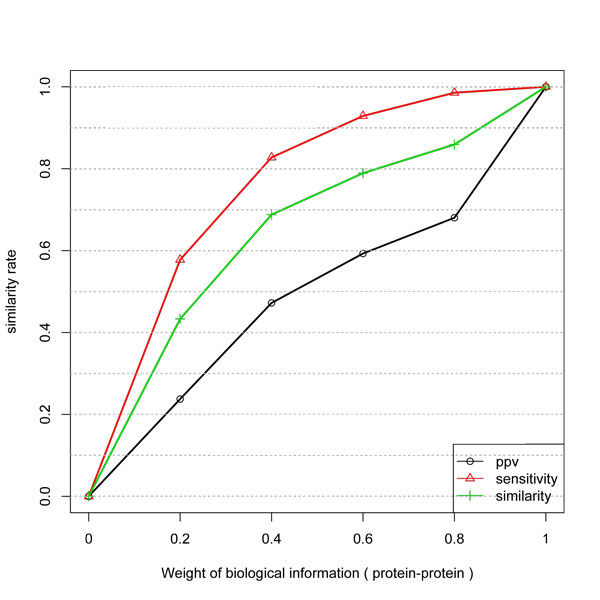
**Similarity, PPV and Sensitivity measures obtained by including different weights of protein-protein information to infer network edges from temporal expression profiles**. The improvement is not linear. In a weight of 0.5 the gain is near 90%.

The choice of *w*_1 _and *w*_2 _is an important problem in data integration for which we performed the experiments presented in Figure 1.

In order to assess the information gain, the experiment was performed starting just with the expression information of the Equation 3, i.e., by setting *w*_1 _= 1 - *w*_2 _and *w*_2 _= 0, 0.2, 0.4, ..., 1.

Figure 1 shows the similarity between the inferred network and protein-protein (gold standard network). It is possible to observe, as expected, that biological information improve the similarity of the network inference method. However, the improvement is not linear and the considered measure present different behavior. The sensitivity measure presents approximately 90% of gain, by setting *w*_1 _= *w*_2 _= 0.5 indicating that protein-protein information is an important data for improvement of GNs inference. It can also be seen that the sensitivity measure presents behavior close to logarithmic. The improvement behavior of the PPV measure indicates that it is a very difficult task due to the complexity of the biological machinery and the indirect relationship between transcripts and proteins.

**Figure 2:**
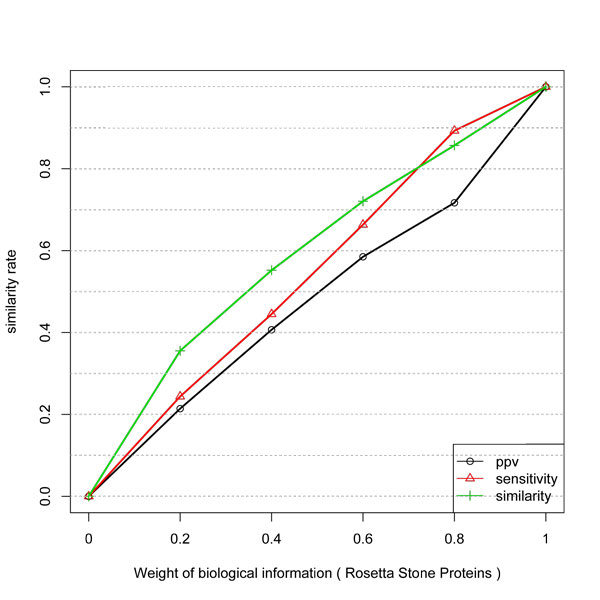
**Similarity, PPV and Sensitivity measures obtained by including different weights of Rosetta Stone information to infer network edges from temporal expression profiles**. Similar to Y2H protein data the gain has a log behavior but PPV has a better gain with Rosetta stone.

Figure 2 shows the result with Rosetta Stone protein linkages network. The behavior of the similarity is not linear. However, PPV presented a linear gain, proportional do the weight of biological information. The same behavior was not observed with protein-protein data of LaCount. A possible explanation is that Rosetta Stone data can have a lower number of False Positives connections among its genes. In other words, a threshold to avoid false positives is considered in the process of selecting putative interactions. Also, only one class of proteins is selected to compose the dataset, i.e., those with separated proteins in an organism and with a fusion of proteins in other species. Therefore, since Y2H is an *in vitro *experiment it can produce a large number of false positive interactions.

**Figure 3:**
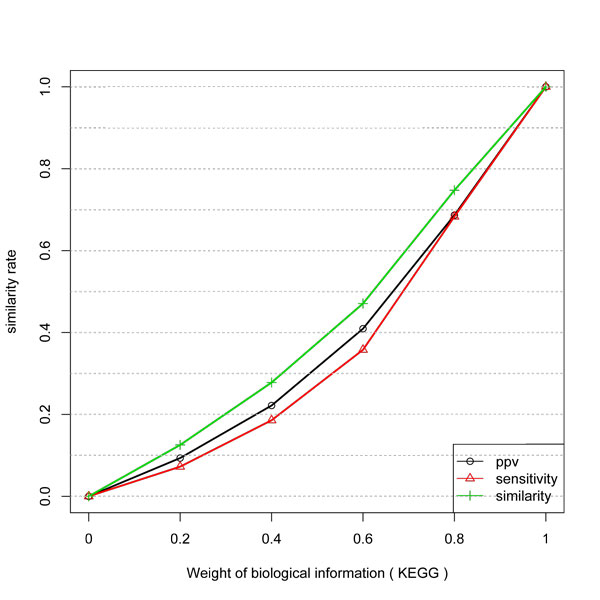
**Similarity, PPV and Sensitivity measures obtained by including different weights of KEGG information to infer network edges from temporal expression profiles**.

**Figure 4:**
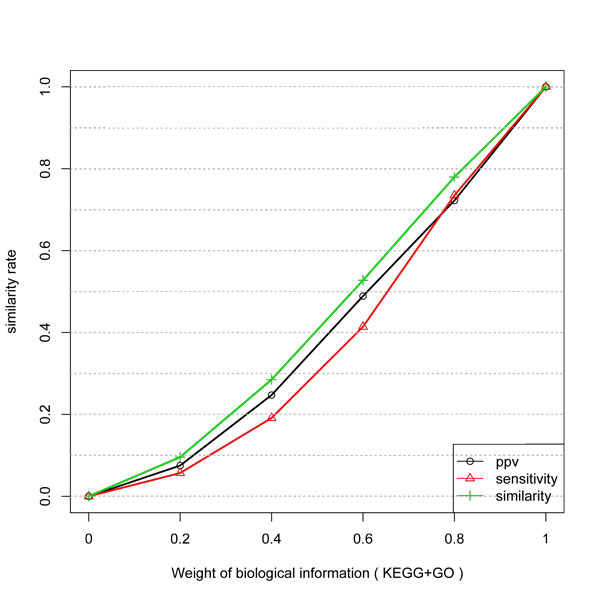
**Similarity, PPV and Sensitivity measures obtained by including different weights of KEGG and GO information to infer network edges from temporal expression profiles**.

The gain of inference with KEGG and Combined KEGG and GO data is presented in Figure 3 and 4. They have a very similar behavior. First, the curves are not linear and not proportional to the weight of the biological information. Also, the gain in PPV is higher than in Specificity. Since KEGG and GO do not guarantee a direct physical interaction between two related genes, some unrelated pairs could be taken as true examples. Also, GO biological process can be more or less specific. Thus the level in the GO graph in which the terms are considered can affect the result since unrelated genes (in a more general process like "metabolism") might be taken as being positive examples.

Thus, the algorithm can predict a direct interaction by considering the biological information weight. By considering the assumption that KEGG and KEGG+GO datasets are true networks, the expression profiles do not match to all its links.

### Evaluation in hub networks

It has been reported that biological networks tend to present some particular topology like scale-free networks. There is expected a few number of proteins with a large number of interactions (hubs) and a large number of proteins with a few number of interactions [2].

**Figure 5:**
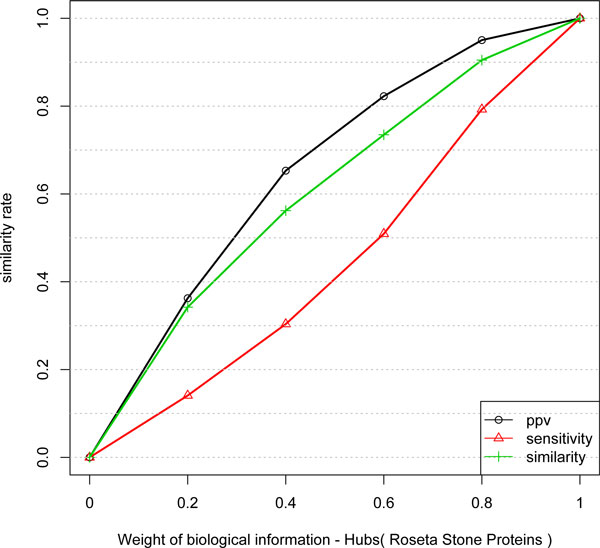
**Similarity, PPV and Sensitivity measures obtained by including different weights of Rosetta stone information to infer the subnetwork of hubs edges from temporal expression profiles**.

**Figure 6:**
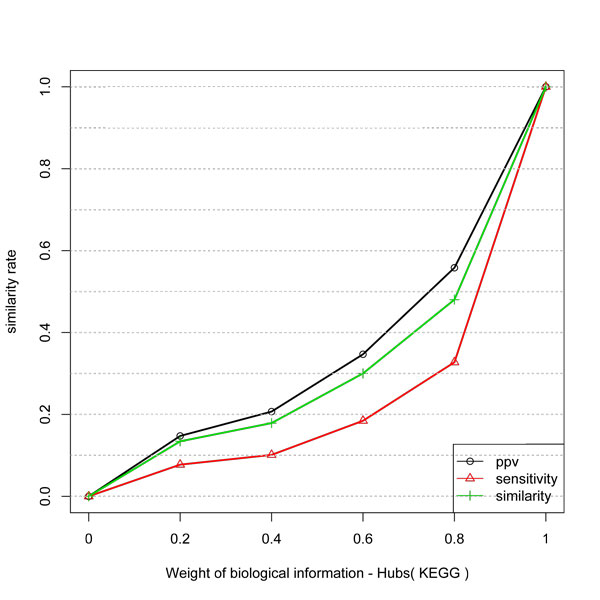
**Similarity, PPV and Sensitivity measures obtained by including different weights of KEGG information to infer the subnetwork of hubs edges from temporal expression profiles**.

Thus, we evaluate the gain by considering a subnetwork composed by the hubs and its interactions. In this study the hubs were defined as the 10% more connected elements in the network. We evaluated the gain for two biological information datasets: Rosetta Stone (direct interaction data) and KEGG (indirect interaction data). The results are shown in Figures 5 and 6. The comparison of the results of Rosetta Stone with the result in Figure 2 shows some interesting behaviors. There is an inversion on the gain between sensitivity and PPV. The Rosetta Stone information improved the gain in PPV for hubs with a logarithmic behavior. For a weight of 0.6 the gain in PPV is near 0.8.

The information of KEGG produced a better improvement in the gain for the global network than for the hub subnetwork. The indirect relationships in this type of data can explain the behavior for the hubs.

### Evaluation in intersection gold standard dataset

**Figure 7:**
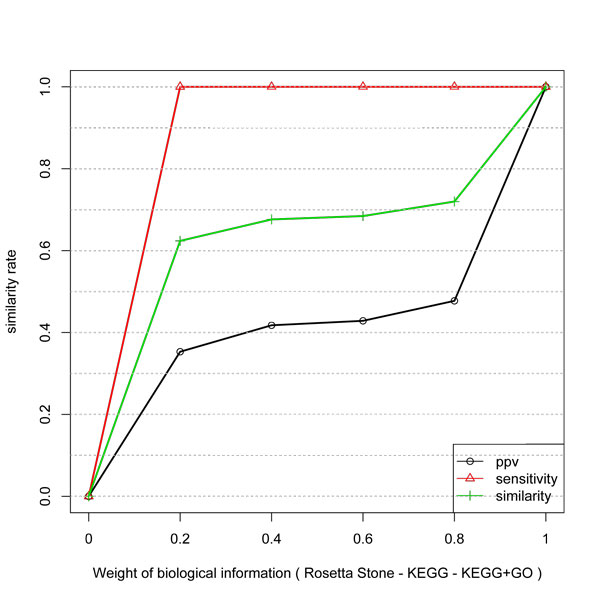
**Similarity, PPV and Sensitivity measures obtained by including different weights of a combined (intersection) network from Rosetta Stone, KEGG and KEGG+GO information to infer the network edges from expression profile**.

**Figure 8:**
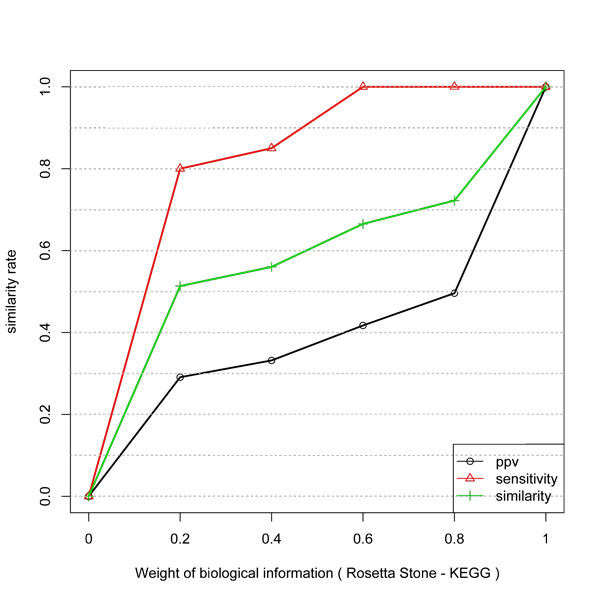
**Similarity, PPV and Sensitivity measures obtained by including different weights of a combined (intersection) network from Rosetta Stone and KEGG information to infer the network edges from expression profile**.

**Figure 9:**
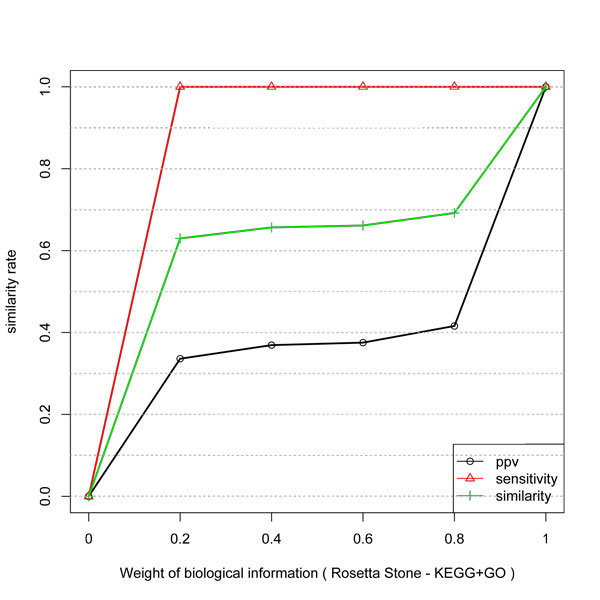
**Similarity, PPV and Sensitivity measures obtained by including different weights of a combined (intersection) network from Rosetta Stone and KEGG+GO information to infer the network edges from expression profile**.

As mentioned before, there is a practical limitation to assembly Intersection Gold Standard datasets. Despite the limitations we assembled the four intersection gold standards presented in Table 3. The IGS1 (Additional files 12 and 13: Tables S12 and S13 respectively), IGS2 (Additional files 14 and 15: Tables S14 and S15 respectively) and IGS3 (Additional files 16 and 17: Tables S16 and S17 respectively) comprises small networks with dozen of vertex and edges. As shown in Figure 7, 8 and 9 the gain is not linear. Although the analyzed intersection networks are a small sampling of the entire network, they present some common features with the original gold standard datasets: (a) they present a non linear behavior in PPV and Sensitivity, (b) the information of Rosetta Stone information seems to maintain a logarithmic behavior even in the intersection to KEGG and GO data.

**Figure 10:**
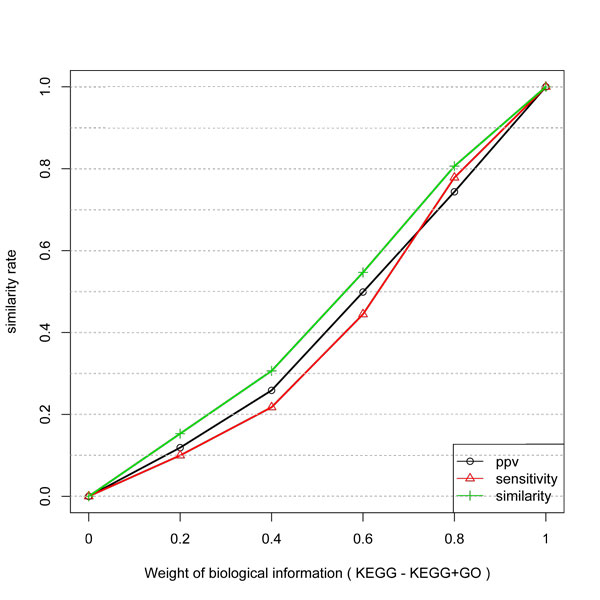
**Similarity, PPV and Sensitivity measures obtained by including different weights of a combined (intersection) network from KEGG and KEGG+GO information to infer the network edges from expression profile**.

The IGS4 comprises a large network with 316 genes and 6, 138 links (Additional files 18 and 19: Tables S18 and S19 respectively). The result of the analysis is shown in Figure 10. A similar behavior with the original gold standard datasets (KEGG and KEGG+GO) can be observed in this analysis. An explanation is that almost the whole original network was maintained (6,138 links of 7,204).

Some interesting features can be observed in the analysis of these data. First, the information of Rosetta stone seems to dominate the behavior when combined to other datasets. In Rosetta stone gold standard (Figure 2) it can be observed that Sensitivity is higher than PPV for any weight. The same behavior can be observed in the intersection datasets of Rosetta Stone (Figures 7, 8 and 9). Second, the curves in intersection datasets containing Rosetta Data present a logarithmic behavior. Third, Rosetta intersection gold standard performance is very similar to protein-protein data and finally. Finally, KEGG and GO present PPV higher than Sensitivity.

These results suggest that data from physical interactions (protein-protein and Rosetta stone) and function or biological process (KEGG and GO) could be classified into two distinct groups with respect to the information gain.

## Conclusion

It is commonly know that data integration is a very important procedure for obtaining better performance on the inference process. However, it is not clear how is the improvement for each information added in the inference method. This work presented an objective way to assess the gain of prediction (PPV and Sensitivity) by adding four types of biological information on an inference method. We also compared the data types and evaluated its relative improvement in the gain.

The results were obtained by applying an inference method of GNs from temporal expression data first obtained from Bozdech [29], filtered, normalized and quantized in [30]. The results indicate, as expected, that adding the biological information data was important for the inference improvement in terms of sensitivity and PPV measures. In particular, the sensitivity measure presented a gain of approximately 90% by setting *w*_1 _= *w*_2 _= 0.5, indicating that protein-protein data improved the recall gain. However, the PPV measure in our experiments indicates that the temporal expression profile has a little contribution to the inference of protein-protein relationships. In addition, one can observe a logarithmic behavior of the sensitivity measure. Also, other biological data like Rosetta stone fusion proteins increases the gain in PPV with an almost linear behavior. The recovering of GNs by using data from KEGG and GO presented a gain that is not proportional to the weight of biological information. This can be explained by the nature of data in which a link between two genes does not mean (ever) a direct interaction. Thus, in this work we suggested that biological information data could be classified into at least two groups: direct interaction (data that informs a physical interaction, verified or predicted) and feature information (data that informs a common feature of the elements like cellular localization, metabolic pathway, etc.). This work represents a starting point to assess how different sources of biological data can be integrated on an inference method and how each information contributes to the improvement of prediction.

In further works, more biological information could be included and a search strategy for the parameters estimation (*w*_i_) could be analyzed for the similarity maximization. Also, integration of data about known unrelated genes (*negative *gold standard) could be tested for the reduction of false positives. Other relevant improvement is to include some different inference methods and biological data sources in the prediction gain analysis. Also, the description about how the level of GO data affects the gain can be another field of investigation.

## List of abbreviations used

FP: false positive; GN: Gene Networks; GO: Gene Ontology; KEGG: Kyoto Encyclopedia of Genes and Genomes; MIPS: Munich Information Center for Protein Sequences; PPV: Positive Predictive Value; SAGE: Serial Analysis of Gene Expression; TP: true positive; TN: true negative; Y2H: Yeast two hybrid.

## Competing interests

The authors declare that they have no competing interests.

## Authors' contributions

FFRV conceived the idea, assembled the datasets, performed the analysis and contributed to drafted the manuscript; FML conceived the idea, developed the computational method and drafted the manuscript; RFH helped in the analysis of the results and contributed to draft the manuscript; RMCJ contributed to the analysis of results and helped to draft the manuscript.

## Supplementary Material

Additional file 1 **USP QC dataset**. USP quality control dataset (leave out oligos with less than 25 samples) Click here for file

Additional file 2 **USP QC dataset samples**. File containing the normalized and quantized signal into three expression levels: -1,0 and 1 (down, null and up regulated). The value 3 means not observed. Click here for file

Additional file 3 **Names of oligos**. Name of each oligo in USP QC dataset samples. Click here for file

Additional file 4 **PIP network**. Protein-Protein Network obtained from LaCount et al, 2005. Click here for file

Additional file 5 **PIP samples**. Samples of the corresponding genes in PIP network. Click here for file

Additional file 6 **Rosetta Stone network**. Rosetta Stone proteins obtained from Date et al, 2006. Click here for file

Additional file 7 **Rosetta Stone samples**. Samples of the corresponding genes in Rosetta Stone network. Click here for file

Additional file 8 **KEGG network**. KEGG gold standard dataset obtained from Bozdech et al, 2003. Click here for file

Additional file 9 **KEGG samples**. Samples of the corresponding genes in KEGG network. Click here for file

Additional file 10 **KEGG and GO network**. KEGG and GO combined gold standard obtained from Date et al, 2006. Click here for file

Additional file 11 **KEGG and GO samples**. Samples of the corresponding genes in KEGG and GO network. Click here for file

Additional file 12 **Intersection Gold Standard Datasets 1 (IGS1)**. Gold Standard network assembled from the intersection between KEGG and KEGG+GO. Click here for file

Additional file 13 **IGS1 samples**. Samples of the corresponding genes in IGS1. Click here for file

Additional file 14 **Intersection Gold Standard Datasets 2 (IGS2)**. Gold Standard network assembled from the intersection between Rosetta Stone and KEGG. Click here for file

Additional file 15 **Intersection Gold Standard Datasets 2 - samples**. Samples of the corresponding genes in IGS2. Click here for file

Additional file 16 **Intersection Gold Standard Datasets 3 (IGS3)**. Gold Standard network assembled from the intersection between Rosetta Stone and KEGG+GO. Click here for file

Additional file 17 **Intersection Gold Standard Datasets 3 - samples**. Samples of the corresponding genes in IGS3. Click here for file

Additional file 18 **Intersection Gold Standard Datasets 4 (IGS4)**. Gold Standard assembled from the intersection between KEGG and KEGG+GO. Click here for file

Additional file 19 **Intersection Gold Standard Datasets 5 - samples**. Samples of the corresponding genes in IGS4. Click here for file
